# Evaluating a novel tool for screening pediatric aspiration and penetration risk: Considerations for safe and effective dysphagia screening in children

**DOI:** 10.1111/dmcn.16424

**Published:** 2025-07-16

**Authors:** Sarah Edney, Katherine Sanchez

**Affiliations:** ^1^ Newcastle University Newcastle upon Tyne UK; ^2^ Protea Therapy Melbourne Australia

## Abstract

**This commentary is on the original article by Cerchiari et al. on pages** 72–81 **of this issue.**

Identifying children at risk for dysphagia is important for prompt treatment initiation and prevention of dysphagia‐related complications; yet there is a lack of proven screening tools appropriate for use with children. Cerchiari et al.^1^ recently published psychometric data for the Pediatric Screening Priority Evaluation Dysphagia (PS‐PED), a screening tool designed to identify dysphagia risk in infants and children. The 14‐item PS‐PED was developed to be user‐friendly for non‐specialists with little or no experience in dysphagia, with each item requiring a yes or no response. Six or more ‘yes’ responses denote ‘at risk’ for dysphagia, while five or fewer ‘yes’ responses are categorized as ‘low risk’. We commend the authors for undertaking the psychometric validation often lacking for dysphagia screening tools^2^ but would like to raise additional considerations for safe and effective dysphagia screening in children.

The first consideration is what condition the PS‐PED screens for. The authors have validated their tool against the Penetration Aspiration Scale,^3^ therefore the PS‐PED screens for risk of airway penetration/aspiration rather than dysphagia. Children with oral phase dysphagia or pharyngeal dysphagia without significant penetration/aspiration may not be identified with this tool, despite also being at increased risk of consequences such as dehydration, malnutrition, restricted growth, and compromised development.^4^

Second, we highlight opportunities to improve the validity and reliability of the PD‐PED via further development of the test items. A number of PS‐PED items are poorly defined and operationalized, compromising the tool's psychometrics, particularly for non‐specialists. For example, the authors could provide further detail on how features such as ‘decreased alertness’ and ‘malnutrition and/or poor growth’ should be defined and measured, how many respiratory tract infections of what type and over what period of time would meet the criteria for ‘recurrent’, and how the appropriateness of a food for a child's developmental stage should be determined. A tool designed to be used by non‐specialists must provide detailed operational definitions and establish item psychometrics to be clinically applicable – and this is particularly pertinent given that the inter‐rater reliability in this study was tested only with speech therapists.

Finally, further consideration should be given to how safely the results of the PS‐PED can be applied and how this may differ across diagnostic groups. The authors note the tool does not account for dysphagia risk being higher in some diagnostic groups than others, and there is no weighting of items. A child with a number of significant risk factors for dysphagia could easily fall below the 6‐point cut‐off. For example, a child with neurological injury affecting the swallowing centres of the brain and recurrent respiratory tract infections should certainly have a specialist dysphagia assessment but may only score ‘2’ on the PS‐PED. The authors acknowledge falling below the PS‐PED cut‐off should not preclude referral for specialist dysphagia assessment; yet unless screening is universal, only children who are already suspected of having dysphagia are likely to be screened. This suggests all screened children should be referred for specialist assessment, regardless of their PS‐PED score. Non‐specialist staff are likely to be falsely reassured by a lower score and take no action, preventing a child from accessing specialist dysphagia assessment and management. In these circumstances, use of the PS‐PED could place children at higher risk than if screening had not taken place at all.

The authors of the PS‐PED acknowledge many of the above concerns and suggest several areas for further development and evaluation of their tool. Given these limitations and the serious consequences of inaccurate dysphagia screening processes, it is reasonable to conclude that the PS‐PED is not yet ready for clinical application. We will watch its further development with interest.

## Data Availability

Not required.
